# Uptake of the antifungal cationic peptide Histatin 5 by *Candida albicans* Ssa2p requires binding to non-conventional sites within the ATPase domain

**DOI:** 10.1111/j.1365-2958.2008.06480.x

**Published:** 2008-10-20

**Authors:** Jianing N Sun, Wansheng Li, Woong Sik Jang, Namrata Nayyar, Mark D Sutton, Mira Edgerton

**Affiliations:** 1 Departments of Oral Biology, School of Dental Medicine, Public Health and Health Professions and Biomedical Sciences, University at BuffaloBuffalo, NY 14214, USA.; 2 Departments of Biostatistics, School of Dental Medicine, Public Health and Health Professions and Biomedical Sciences, University at BuffaloBuffalo, NY 14214, USA.; 3 Departments of Biochemistry, School of Dental Medicine, Public Health and Health Professions and Biomedical Sciences, University at BuffaloBuffalo, NY 14214, USA.; 4 Departments of Restorative Dentistry, School of Dental Medicine, Public Health and Health Professions and Biomedical Sciences, University at BuffaloBuffalo, NY 14214, USA.

## Abstract

*Candida albicans* Hsp70 Ssa1/2 proteins have been identified as cell wall binding partners for the antifungal cationic peptide Histatin 5 (Hst 5) *in vivo*. *C. albicans* Ssa2p plays a major role in binding and translocation of Hst 5 into fungal cells, as demonstrated by defective peptide uptake and killing in *C. albicans SSA2* null mutants. Candidal Hsp70 proteins are classical chaperone proteins with two discrete functional domains consisting of peptide binding and ATP binding regions. Pull-down assays with full-length and truncated Ssa2 proteins found that the ATPase domain was required for Hst 5 binding. Further mapping of Ssa2p by limited digestion and peptide array analyses identified two discrete Hst 5-binding epitopes within the ATPase region. Expression of Ssa2p in *C. albicans* cells carrying mutations in the first epitope identified by thermolysin digestion (Ssa2_128−132A3_) significantly reduced intracellular transport and fungicidal activity of Hst 5, confirming its importance as a binding site for Hst 5 function *in vivo*. Since this Hst 5 binding site lies within the Ssa2p ATPase domain near the ATP-binding cleft, it is possible that ATP modulates Hst 5 binding to Ssa2p. Indeed, gel filtration assays demonstrated that although nucleotides are not required for Hst 5 binding, their presence improved binding affinity by 10-fold. Thus, *C. albicans* Ssa2p binds Hst 5 at a surface-localized epitope in a subunit of the ATPase domain; and this region is required for intracellular translocation and killing functions of Hst 5.

## Introduction

Histatin 5 (Hst 5) is a histidine-rich, antifungal cationic protein (24 amino acids) secreted by the major salivary glands only in humans and higher primates. Hst 5 is strongly fungicidal for *Candida albicans* and other fungal pathogens associated with oral candidiasis. Unlike other cationic peptides, the fungicidal mechanism of Hst 5 is not a result of cytolysis or membrane disruption. Instead, Hst 5 induces selective leakage of intracellular ions and ATP from yeast cells resulting in gradual cell death that is similar to osmotically induced cell death (Koshlukova *et al*., 2000; Vylkova *et al*., 2007). These cytotoxic effects are initiated once Hst 5 reaches the intracellular compartment; therefore the ability of this protein to be transported into the cell is essential for its fungicidal activity. We found that *C. albicans* expresses cell wall proteins that bind Hst 5, which we identified as Heat Shock Protein (Hsp) 70 family members Ssa1 (656 amino acids) and Ssa2 (645 amino acids) (Li *et al*., 2003). Ssa2 protein is more important than Ssa1 in mediating toxicity of Hst 5, since Hst 5 intracelluar uptake and killing are significantly reduced in *C. albicans SSA2* deletion mutants while *SSA1* knockouts are only mildly resistant to Hst 5 (Li *et al*., 2006). This unexpected role of Ssa2 protein in facilitating intracellular translocation of Hst 5 prompted us to examine the molecular basis for its interactions with Hst 5.

Ssa (stress seventy a subfamily) proteins belong to the Hsp70 family, of which *Candida* has only two members: Ssa1p and Ssa2p. Ssa proteins in *Candida* are predominantly localizated in the cytoplasm (Li *et al*., 2006) where they function as chaperones in the translocation of client proteins across microsomal membranes (Ngosuwan *et al*., 2003) and transportation of proteins to the vacuole for degradation (Brown *et al*., 2000; Satyanarayana *et al*., 2000). However, both *C. albicans* and *Saccharomyces cerevisiae* export Hsp70 proteins, including Ssa1p and Ssa2p, to the cell wall (Lopez-Ribot and Chaffin, 1996; Lopez-Ribot *et al*., 1996; Nombela *et al*., 2006). These proteins are likely directed to the cell surface through a non-conventional secretion pathway, since yeast Ssa proteins do not contain an N-terminal secretion signal (Nombela *et al*., 2006). In contrast to cytosolic Ssa proteins, the general function of cell wall-localized Ssa proteins is not known, although our studies suggest that they may have binding activity with extracellular proteins.

All Hsp70 proteins contain two functional regions, an ATPase domain and peptide-binding domain, which interplay in the process of binding and release of client substrates. The larger ATPase domain is composed of two equally sized lobes (designated I and II) that are separated by a deep cleft containing the nucleotide binding site (Flaherty *et al*., 1990). Each lobe can be further subdivided into two separate topological domains IA, IIA, IB and IIB of which the IA (∼110 residues) and IIA (∼130 residues) subunits form the nucleotide-binding cleft. Conventional protein binding with yeast cytosolic Ssa chaperones requires specific sites within its C-terminal peptide-binding domain, and is driven by cycled ATP binding and hydrolysis within the N-terminal domain (Mayer *et al*., 2000). However, it is not known whether binding with smaller peptides of non-fungal origin (such as Hst 5) involves the same nucleotide-dependent substrate-binding process. Since ATP levels within the cell wall are likely to be low under normal conditions, it is possible that binding of non-native peptides by cell wall Ssa proteins is largely ATP independent and may occur at non-conventional binding sites. Indeed, evidence for such atypical binding functions by microbial Hsp70 proteins is accumulating. Surface-localized Hsp70 proteins in *Heliobacter pylori* and *Hemophilus influenza* have binding specificity for sulfogalactolipids consistent with a cell surface receptor function which was mapped to the ATPase domain (Mamelak *et al*., 2001). Bacterial periplasmic surface-localized Hsp70 (DnaK) is actively involved in transport of peptides and prevents aggregation of several unfolded proteins (Richarme and Caldas, 1997). Similar to our findings with candidal Ssa proteins, binding by bacterial DnaK to cationic antimicrobial peptides including drosocin, apidaecin and pyrrhocoricin was shown to be related to their intracellular uptake and killing functions (Kragol *et al*., 2001). The probable interaction sites between DnaK and pyrrhocoricin were mapped to the C-terminal variable region of the lid domain outside the conventional peptide binding region (Otvos *et al*., 2000; Kragol *et al*., 2001). Thus, microbial cell surface-localized Hsp70 proteins appear to have a broad role in recognition and binding of substrate proteins, and may utilize sites other than the conventional peptide binding region described for cytosolic family members.

To determine whether Hst 5 binding with *C. albicans* Ssa2 protein utilizes the conventional nucleotide-dependent peptide-binding domain or instead involves other novel binding sites, we mapped Hst 5-binding epitopes using immunoprecipitation, limited digestion and peptide array strategies. Here we report that Hst 5 binding maps to the IA subunit region in the ATPase domain of Ssa2p, and *in vivo* expression of *C. albicans* Ssa2p carrying mutations within this identified site reduced binding and intracellular uptake of Hst 5 independently of other co-chaperones and nucleotides. Thus binding of yeast Ssa2 protein with human salivary Hst 5 involves at least one epitope (Ssa2_128−132_) within the ATPase region.

## Results

### Ssa2p ATPase domain (Ssa2_1−385_) binds Hst 5

**Fig. 1:**
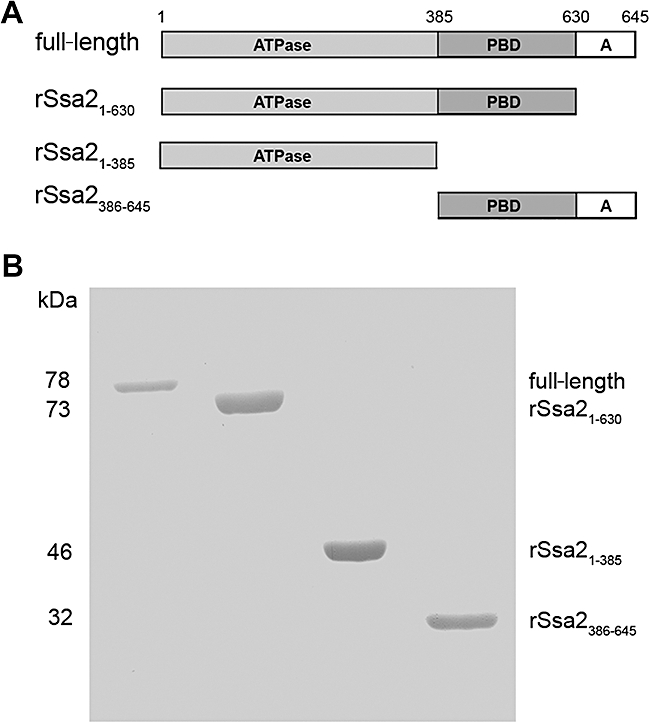
Expression and purification of full-length and truncated Ssa2 proteins. A. Schematic representation of the domain structure of *Candida albicans* Ssa2p and the design for truncated Ssa2 proteins. B. Each purified recombinant protein obtained from a yeast expression system (1 μg) was subjected to 10% SDS-PAGE and Coomassie blue-stained to visualize *C. albicans* full-length, rSsa2_1−630_, rSsa2_1−385_, rSsa2_386−645_ proteins.

Since *C. albicans* Ssa2p has stronger association with Hst 5 than Ssa1p (Li *et al*., 2006), we used this Hsp70 family member for mapping Hst 5-binding domains. A series of truncated *C. albicans* Ssa2 proteins were constructed (Fig. 1A) guided by known functional domain structures of *S. cerevisiae* Ssa1p (Qian *et al*., 2002). Recombinant Ssa2 proteins encompassing the ATPase domain (rSsa2_1−385_), the peptide-binding domain (rSsa2_386−645_), and full-length Ssa2 protein deleted of 15 amino acid residues of the anchor region at the C-terminus (rSsa2_1−630_) were constructed and expressed in a yeast expression system. Each purified protein (> 95%) was subjected to SDS-PAGE and Coomassie blue-stained to verify that the apparent molecular weight of each expressed protein corresponded to its predicted size (Fig. 1B).

**Fig. 2:**
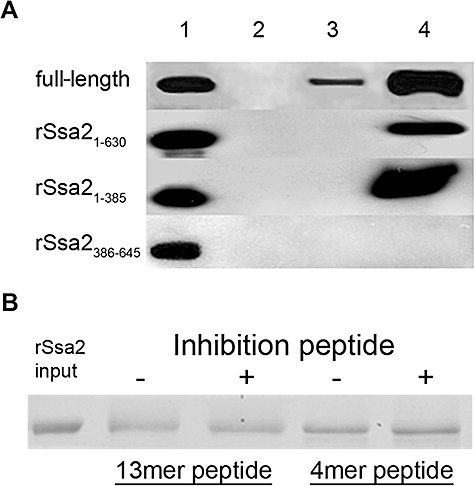
ATPase domain of Ssa2 is necessary for complex formation with BHst 5. A. Purified full length, rSsa2_1−630_, rSsa2_1−385_ or rSsa2_386−645_ (input) were incubated with Biotin-Hst 5 (BHst 5) for 2 h at 4°C with or without the presence of cross-linker to allow complex formation, and Streptavidin-agarose (SA) beads were added to the mixture. Resulting complexes were isolated by centrifugation of the SA beads and washed to remove non-specifically bound proteins. Recovered protein complexes were subjected to SDS-PAGE and detected by Western blotting with anti-Xpress-HRP monoclonal antibody and enhanced chemiluminescence (ECL). Lane 1: 10% of input Ssa2 proteins; lane 2: negative control, pull-down Ssa2 proteins without BHst 5; lane 3: pull-down Ssa2 proteins with BHst 5 without cross-linker; lane 4: pull-down Ssa2 proteins by BHst 5 with cross-linker*.* Complex formation was detected only with proteins containing the ATPase domain. B. A six fold molar excess of Ssa2p C-terminal anchor domain peptide 13mer (EPSNDGPTVEEVD) or 4mer (EEVD) was pre-incubated with BHst 5 for 30 min at 4°C prior to addition of full-length rSsa2p for the pull-down assay described in (A). No inhibition of interactions between Ssa2p and Hst 5 was observed with either peptide (+) compared with Hst 5 and Ssa2p alone (−).

To determine which domains are essential for interaction with Hst 5, BHst 5 (Biotin-Hst 5) was used as the bait protein in pull-down assays with Ssa2 proteins in native as well as chemically cross-linked conditions as previously described (Li *et al*., 2006). Although binding complexes were detected between BHst 5 and full-length rSsa2p in pull-down assays in native conditions, none of the truncated Ssa2 variants could form complexes with BHst 5 (Fig. 2A, lane 3), indicating that full-length Ssa2p is optimal for formation of stable complexes with BHst 5 in these assays. Addition of a cross-linker to stabilize interactions resulted in detection of BHst 5 complexes with both rSsa2_1−630_ and rSsa2_1−385_ (Fig. 2A, lane 4); however, Ssa2p variant missing the ATPase domain (rSsa2_386−645_) did not form any detectable complexes with BHst 5. To rule out the possibility that the cross-linker itself contributed to the pull-down complex, truncated Ssa2 proteins were incubated with cross-linker and Streptavidin conjugated beads; however, no complex formation was detected (data not shown). Thus, the 46 kDa ATPase domain of Ssa2 (rSsa2_1−385_) appeared to be the crucial domain involved in binding of Hst 5, consistent with its reported function of binding nucleotides and accessory proteins such as Hsp40, Hip, Hop, Hap and BAG-1 (Benjamin and McMillan, 1998).

Since the C-terminus of Hsp70 contains docking sites for co-chaperones Hsp40 and Hsp90 (Qian *et al*., 2002; Carrigan *et al*., 2006), we constructed an Ssa2p truncated in this classical protein anchor domain (rSsa2_1−630_). However, Ssa2p deleted of this anchor region still was able to associate with BHst 5, showing that this anchor domain is not required for binding with Hst 5 (Fig. 2A). To verify this result, two peptides representing the Ssa2_631−645_ anchor domain (EPSNDGPTVEEVD), as well as a 4mer peptide EEVD recognition sequence from this domain were tested for their ability to inhibit complex formation between full-length rSsa2p and BHst 5. Neither the anchor domain protein nor the EEVD peptide was able to inhibit rSsa2p–BHst 5 complex formation when pre-incubated with BHst 5 as assessed by pull-down assays (Fig. 2B). Thus, neither of these classical peptide anchor regions appears to be required for Hst 5 binding with Ssa2p, although it is possible that they may contribute to structural features of Ssa2p involved in substrate binding.

### Two epitopes within the Ssa2p ATPase domain are putative binding sites for Hst 5

**Fig. 3:**
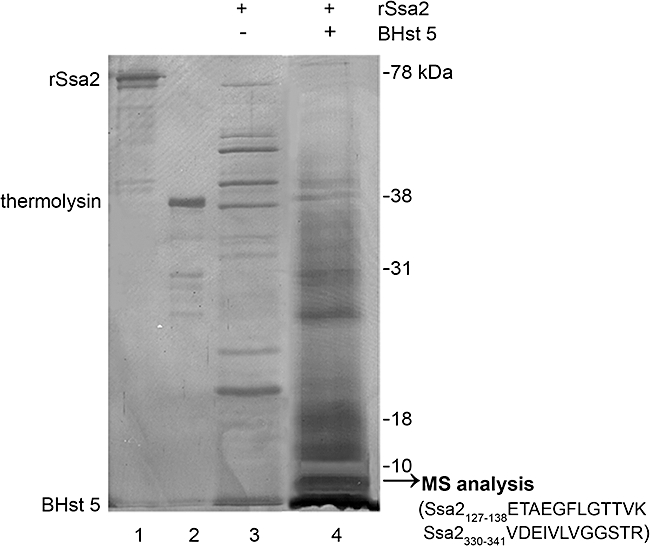
Digestion products of rSsa2p are substantially altered by BHst 5 binding. Purified rSsa2p (10 μg) was incubated with 20 μg of BHst 5 in the presence of 2 mM ATP-Mg^2+^ and cross-linker for 30 min at room temperature. Stabilized rSsa2p–BHst 5 complexes were isolated by SA beads pull-down. The complexes was then subjected to 5 μg ml^−1^ thermolysin digestion at 30°C for 1 h. Digestion fragments not associated with the complex binding site were removed by washing, and the remaining bead-retained rSsa2p–BHst 5 complex was recovered and half was subjected to 15% SDS-PAGE and detected by silver staining (lane 4). Ssa2p digestion products differed quite substantially in the presence of Hst 5. Two peptides within the ATPase domain were identified by MS analysis of a digestion fragment (arrow). Lane 1: rSsa2p (0.5 μg); lane 2: thermolysin (0.5 μg); lane 3: thermolysin digestion of rSsa2p without BHst 5; lane 4: thermolysin digestion products from rSsa2p–BHst 5 complex.

**Fig. 5:**
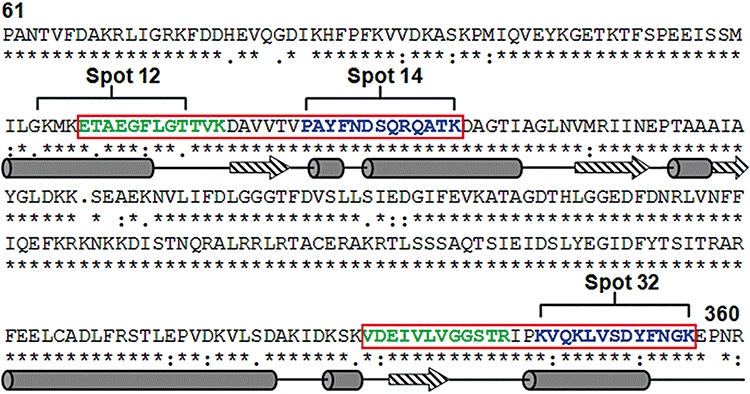
BHst 5-binding epitopes of Ssa2p are localized within the ATPase domain. The deduced primary sequences of Ssa1p and Ssa2p from *C. albicans* were aligned to show conserved regions (*) and variable regions (: or .). Hst 5 binding sites on Ssa2p identified by limited digestion (green) and peptide array (blue) (spots 12, 14 and 32) are indicated, and contiguous regions are enclosed by red boxes. Predicted secondary structure of BHst 5 binding regions was shown as α-helices (cylinders) and β-strands (arrows) below the primary sequences.

To more finely map Hst 5 binding sites within the rSsa2p ATPase domain, limited digestion of cross-linked rSsa2p–BHst 5 complexes was performed. Thermolysin was selected as the proteolytic enzyme for these experiments since Hst 5 contains only one weak digestion site at its extreme N-terminus, while Ssa2p contains over 30 sites distributed over its 645 amino acid length (http://www.bioinf.manchester.ac.uk/nickpred/). Following enzymatic digestion of Ssa2p–Hst 5 cross-linked complexes, Ssa2p digestion fragments not associated with BHst 5 were removed by washing, while those associated with BHst 5 were recovered by pull-down with Streptavidin beads and separated by 15% SDS-PAGE (Fig. 3, lane 4). Same amount of Ssa2p was digested with thermolysin without the presence of BHst 5 as a control and loaded directly on the SDS-PAGE gel (Fig. 3, lane 3). Strikingly different digestion products of rSsa2p were observed when rSsa2p was bound to BHst 5 (Fig. 3, lane 3 and lane 4 respectively), showing that Hst 5 binding substantially alters available thermolysin digestion sites exposed for enzymatic cleavage. The smallest major digestion fragments (≈ 8 kDa) released from Hst 5–Ssa2p complexes were selected for analysis by mass spectrometry (Fig. 3, lane 4, arrow) as being representative of more complete digestion products of flanking regions. Higher-molecular-weight digestion products were not further characterized since they were likely to contain regions more distally removed from cross-linked binding sites. Two peptides were identified: ETAEGFLGTTVK (Ssa2_127−138_) and VDEIVLVGGSTR (Ssa2_330−341_), both of which are located in the ATPase domain of Ssa2p, but are separated by 172 amino acid residues (Fig. 5, green regions). These results confirmed that the ATPase domain is indeed the site for binding with Hst 5, but suggested the possibility of two independent binding sites.

### Hst 5-binding motifs within the Ssa2p ATPase domain

**Fig. 4:**
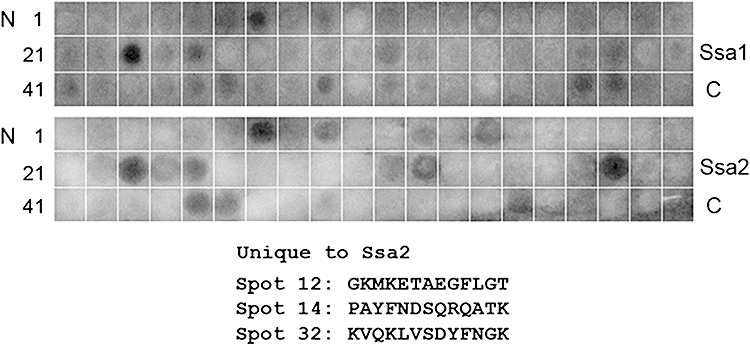
BHst 5 binds with epitopes in Ssa1 and 2 peptide arrays. BHst 5 (2 μM) was incubated with Ssa1 or Ssa2 peptide array membranes for 3 h at room temperature. After extensive washing with TBS buffer to remove unbound BHst 5, the membrane was incubated with Streptavidin-HRP (1:20 000) for 1 h at room temperature and the image developed with SuperSignal West Pico Chemiluminescence Substrate. Hst 5 binding to peptides sequences unique to Ssa2 protein is shown.

In order to assess binding epitopes by an alternative approach as well as to compare relative binding of Hst 5 with Ssa2p and Ssa1p, we used scanning peptide arrays of Ssa1p concurrently with Ssa2p. Peptide arrays provide a high-throughput method for simultaneous measurement of binding to discrete peptides under the same binding conditions. Peptide arrays containing a complete library of Ssa1 and Ssa2 proteins were incubated with BHst 5 (2 μM), then with Streptavidin-HRP and quantified following ECL development (Fig. 4). Among the strongest peptide binding sites for Hst 5, three spots (7, 23 and 38) as well as four less intense spots (9, 25, 45, 46) were identified in common to both Ssa1 and Ssa2 arrays (Fig. 4). These peptides sequences are nearly identical in both Ssa1p and Ssa2p (*Supporting information*, Data S1), therefore we did not pursue them further as they were not likely to show a specific basis for binding of Hst 5 with Ssa2p. Also, we observed that these three peptides showed a high degree of homology in primary sequence with Hst 5 suggesting self-self binding, since previously we had found that Hst 5 has a high propensity for self-aggregation (Li *et al*., 2006).

Interestingly, three peptides were identified (spots 12, 14 and 32) that exhibited strong binding with BHst 5 (and without homology to Hst 5) as well as being unique for Ssa2p. Importantly, these peptides were located within the ATPase domain of Ssa2p protein and either overlapped, or were adjacent to fragments identified by limited thermolysin digestion. One other unique Ssa2p-binding epitope (spot 55) was identified but not further examined since it was outside the ATPase domain. Two distinct Hst 5 binding regions formed by contiguous epitopes identified by these two independent methods (marked with red boxes Fig. 5) were mapped on primary and secondary structures of *C. albicans* Ssa2p. Both regions were found to have defined secondary structures consisting of adjacent β-sheet and α-helical regions, and have numerous non-conserved amino acids (Fig. 5), potentially providing the basis for selectivity of Hst 5 binding with Ssa2p.

### Mutagenesis of *C. albicans* Ssa2p–Hst 5 T1-binding epitopes reduces function

We next examined whether the epitopes identified from *in vitro* binding assays would have a functional role for Hst 5 binding in living cells. As a first approach, synthetic peptides identified as binding epitopes (T1: Ssa2_127−157_, T2: Ssa2_329−356_, and peptide array spots Ssa2_68−83_ and Ssa2_245−257_) were used for competition experiments in which Hst 5 and each peptide were pre-incubated together in solution before addition to *C. albicans* cells. However none of the peptides significantly reduced Hst 5 killing and translocation, showing that Hst 5 interactions with Ssa proteins do not take place at extracellular regions and/or do not occur with small linear fragments of Ssa2p. Therefore, our next approach was to use site-directed mutagenesis of each epitope in full-length Ssa2p *in vivo*.

**Fig. 6:**
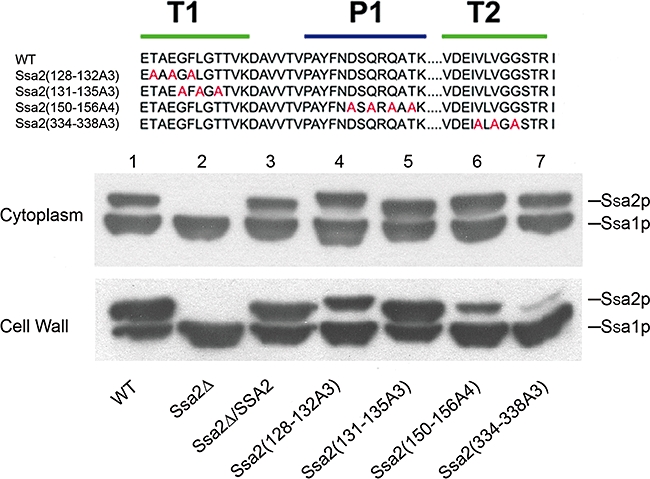
Design and expression of mutated Ssa2 proteins in *C. albicans* strains. Regions selected for site-directed mutagenesis within Hst 5 binding regions are based upon results of thermolysin limited digestion (green bars, also indicated as T1 and T2 regions) and peptide array (blue bar, also indicated as P1 region). Amino acids selected for Ala substitution are shown in red (top). Cell wall and cytoplasmic proteins were isolated from each strain (bottom): 25 μg of each protein was loaded on 7.5% SDS-PAGE gel and immunoblotted with Hsp70/Hsc70 monoclonal antibody. *C. albicans* wt cells display two distinct Hsp70 proteins – Ssa1p (lane 1, lower band) and Ssa2p (lane 1, upper band). Only Ssa1p is expressed in the *ssa2*Δ knockout (lower band, lane 2), while the gene restoration strain (*ssa2*Δ*/SSA2*) expressed both Ssa2p and Ssa1p comparable with wt cells (lane 3). Gene restoration constructs of *SSA2* with three to four amino acid substitutions (lane 4 to lane 7) expressed similar amounts of Ssa2 proteins as wt. However, significantly less mutant Ssa2_(334−338A3)_ protein was observed in cell wall fraction (lane 7, bottom).

A series of Ssa2p mutagenesis sites were designed within thermolysin digestion fragments (Fig. 6, top, T1 and T2 regions shown beneath green bars); and within spot 14 from the peptide array (P1 indicated with a blue bar). Alternating amino acids were mutated using mutagenesis primers and were integrated into the *ssa2*Δ strain genome. We expected that strains expressing mutations within a region unrelated to Hst 5 functions would be similar to the gene restoration strain *ssa2*Δ*/SSA2* that expresses wild-type (wt) protein*;* while mutations within regions essential for Hst 5 binding would have a phenotype like the *ssa2*Δ null mutant. Cytosolic expression levels of all mutated Ssa2 proteins (Fig. 6, lanes 4–7) were very similar to wt (Fig. 6, lane 1) and gene restoration *ssa2*Δ*/SSA2* (Fig. 6, lane 3) strains. Mutated Ssa2 proteins from T1 and P1 regions were secreted to the cell wall at levels comparable to wt protein (Fig. 6, bottom, lanes 4–6); however, mutation of only three amino acid residues within the T2 region of protein Ssa2_(334−338A3)_ nearly abolished its secretion to the cell wall (Fig. 6, lane 7). Thus we expected attenuation of Hst 5 translocation in this strain as a result of this secretion defect independently of amino acid substitutions.

**Fig. 7:**
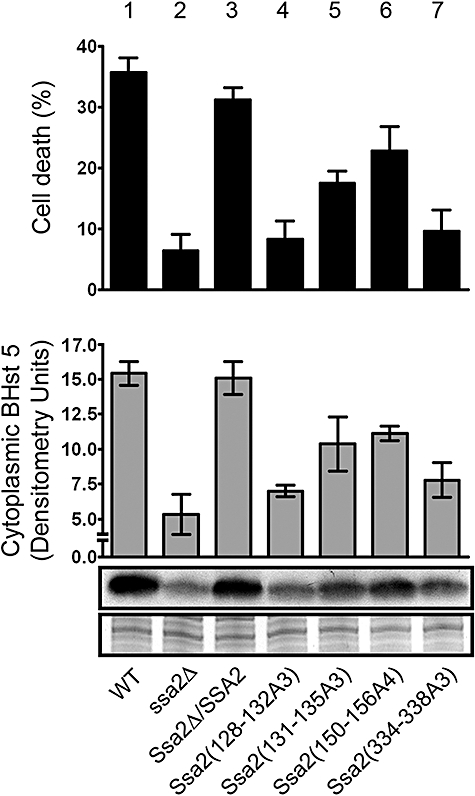
Mutations in Ssa2p Hst 5-binding epitopes reduce Hst 5 uptake and cytotoxicity. *C. albicans* constructs of *SSA2* mutations were tested for sensitivity to Hst 5 (top) and ability to translocate Hst 5 to the cytosol (bottom). Candidacidal assays were performed by incubating cells with 15 μM Hst 5 for 1 h at 30°C, and percentage cell death was calculated compared with untreated cells. Cytosolic translocation of Biotin-Hst 5 (15 μM) was measured for each *C. albicans* construct using the same conditions as for candidacidal assays. Cytosolic proteins (10 μg) from each construct were subjected to 16% Tricine SDS-PAGE, immunoblotted with Streptavidin-HRP to detect BHst 5, and quantified. Control proteins from each construct are shown to verify equal protein loading. Mutations in Ssa2_(128−132A3)_ resulted in complete loss of killing and translocation (lane 4) equivalent to the *ssa2*Δ knockout, while mutations in Ssa2_(334−338A3)_ (lane 7) had significant loss of cytotoxic and transport functions. Mutations in Ssa2_(131−135A3)_ and Ssa2_(150−156A4)_ (lane 5 and lane 6) resulted in mild to moderate loss of function.

Next, strains carrying Ssa2p mutations were compared with wt and *ssa2*Δ*/SSA2* gene restoration strains for their ability to translocate Hst 5 and for cell susceptibility to Hst 5 cytotoxicity (Fig. 7). Mutations within the first T1 region in Ssa2_(128−132A3)_ resulted in the most pronounced reduction of Hst 5 intracellular translocation and toxicity (Fig. 7, lane 4) to levels of *ssa2*Δ knockout cells (Fig. 7, lane 2). Thus, the TAEGF epitope (found only in Ssa2p) is crucial for Hst 5 activity and may be one reason for propensity of Hst 5 binding for Ssa2p over Ssa1p. These results also confirm involvement in this region for Hst 5 binding as determined by *in vitro* thermolysin digestion experiments. In contrast, mutations in the downstream T1 region of Ssa2p (131–135) reduced, but did not prevent Hst 5 killing and intracellular uptake (Fig. 7, lane 5), suggesting that this region may partially overlap the Hst 5 binding site or may modulate Ssa2p conformation required for binding. As expected, the T2 Ssa2_(334−338A3)_ mutant with defective cell wall secretion (Fig. 7, lane 7) had reduced Hst 5 translocation and killing to levels of the *ssa2*Δ knockout. Attempts to produce substitution mutations within other regions of T2 failed, resulting in complete loss of both cytosolic and cell wall Ssa2 protein. Thus, this region appears to be critically important for correct protein expression as well as its secretion to the cell wall. However, we could not validate its ability to interact with Hst 5 using a mutational approach. Mutation of the P1 region of Ssa2_(150−156A3)_ resulted in approximately 20% reduction in Hst 5 translocation and in cellular killing (Fig. 7, lane 6), which may be a consequence of a slight reduction in cell wall secretion of this mutated protein (Fig. 6, lane 6). Similarly, mutations within the region of Ssa2_(342–355)_ representing spot 32 in the peptide array did not alter Hst 5 killing or translocation (data not shown), thus showing that this region is not involved in Hst 5 function. Thus, Hst 5 binding regions identified using linear probes in the peptide array do not appear to be actual binding sites *in vivo*, but may have some secondary role.

**Fig. 8:**
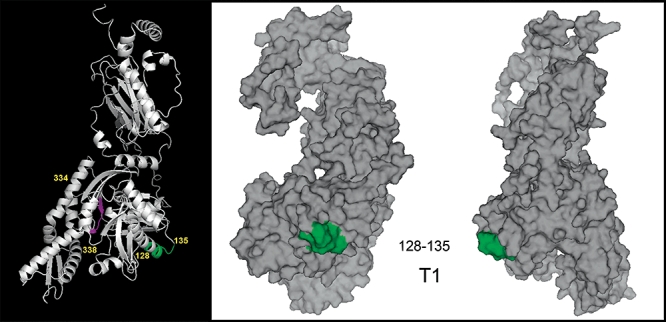
Hst 5 binding sites within Ssa2p lie near the nucleotide-binding cleft of the ATPase domain. Tertiary structure of full-length *C. albicans* Ssa2p was predicted from ROBETTA full-chain protein structure Prediction Server. An epitope found to strongly affect Hst 5 function is labelled with green (Ssa2_128−135_), and a region affecting Hst 5 function as well as cell wall secretion (Ssa2_334−338_) is shown in magenta. Ssa2p tertiary structure was modelled using PyMOL software and is shown as a ribbon structure (left) and as a surface model (centre and right). The Hst 5 binding site Ssa2_128−135_ is located on the ‘shoulder’ of the nucleotide-binding cleft of the IA subunit of the ATPase domain and is completely surface accessible (centre and right), while Ssa2_334−338_ lies in the nucleotide-binding cleft (left) and is not surface accessible.

In order to visualize the spatial relationship and surface accessibility of sites identified as important for Hst 5 activity by mutational analyses, these epitopes were mapped on a tertiary model of full-length *C. albicans* Ssa2p predicated using ROBETTA Full-chain Protein Structure Prediction Server (Fig. 8). The T1 Hst 5 binding site identified by *in vivo* assays with the highest function effects was labelled with green (Ssa2_128−135_) and the T2 region with possible functional effects was labelled with magenta (Ssa2_334−338_). Ribbon structure mapping (Fig. 8, left) showed that the strong Hst 5 interaction site (green) is located on the ‘shoulder’ of the ATPase domain within the IA subunit lobe. Surface modelling of this site (Fig. 8, centre and right) showed that this area is quite surface accessible. In contrast, the intermediate functional region (magenta) lies within the ATP-binding cleft of the IIA subunit and has little surface accessibility. Thus changes in Hst 5 binding upon mutation of this region may be a result of structural modification, or loss of the ability of ATP to bind the groove area in the ATPase domain. This raised the question whether Hst 5 binding is influenced by conformational changes of Ssa2p as a result of functional binding with ATP or other nucleotides as a part of its typical chaperone function.

### Hst 5 does not compete for nucleotide binding sites in Ssa2p

**Fig. 9:**
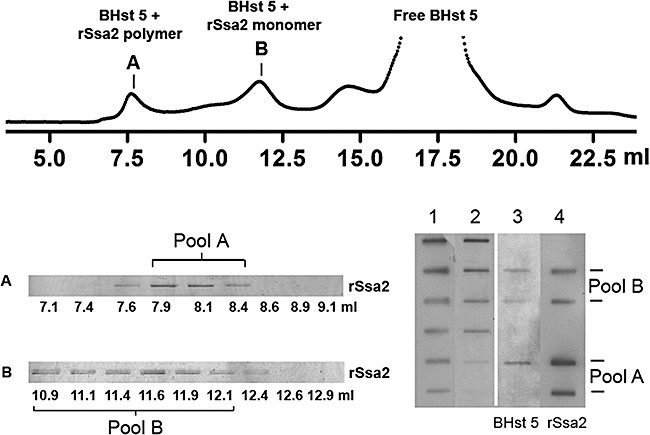
BHst 5 co-elutes with rSsa2p. Purified rSsa2p (5 μM) was mixed with excess BHst 5 (100 μM) for 30 min before loading onto Superose column. Fractions were collected (top) and subjected to 7.5% SDS-PAGE and silver-stained to detect rSsa2p (bottom left). rSsa2p polymer peak and monomer peak fractions were pooled to generate pool A (∼640 μl) and pool B (∼1300 μl) respectively. Both co-eluted BHst 5 and rSsa2 proteins from each pool were quantified by slot blot with corresponding linear loading standards (bottom right). Two slots of each pool were loaded (lane 4) corresponding to 25% and 5% of total volume in order to be within the linear range for quantification. Lane 1: rSsa2p loading standards (300, 150, 75, 37.5, 18.8, 9.4, 4.7 ng); lane 2: BHst 5 loading standards: (125, 100, 50, 25, 12.5, 6.3 ng); lane 3: BHst 5 co-eluted in pool A or pool B was quantified following detection by Streptavidin-HRP antibody; lane 4: quantification of rSsa2p was performed on the same membrane after being stripped and re-probed with Anti-Xpress antibody to detect rSsa2p. Co-eluted BHst 5 and rSsa2p under nucleotide treatment conditions were similarly quantified.

Gel filtration analyses were used to assess binding of BHst 5 to rSsa2p in the presence of nucleotides. The elution profile for purified rSsa2p alone was quite similar to previously reported data (Gao *et al*., 1995), and identified both Ssa2p monomer (eluted at 11.5 ml) and polymer (eluted at 7.5 ml) peaks well separated from the free BHst 5 peak (17.5 ml) (Fig. 9). A 10-fold molar excess of BHst 5 was incubated with rSsa2p for 30 min with either 1 mM ATP-Mg^2+^ 1 mM ATPγS-Mg^2+^ or 0.9 mM ADP-Mg^2+^, 0.1 mM ATP-Mg^2+^ and 1 mM P_i_ to allow complex formation, then subjected to gel filtration using a pre-equilibrated column in Buffer A containing nucleotides. Eluted rSsa2p–BHst 5 complexes (both monomeric and polymeric rSsa2p) were collected, subjected to SDS-PAGE to detect rSsa2p, and quantified by slot blot analyses (Fig. 9). Since these two proteins are of different sizes (645 amino acids and 24 amino acids respectively) which could result in differences in blotting efficiency, we first optimized loading to produce linear standards (Fig. 9, right, lanes 1 and 2) and then selected an appropriate volume from each pool in order to quantify each protein within its linear range. As expected, BHst 5 was co-eluted with both the rSsa2p monomer and rSsa2p polymer peaks, although the majority of BHst 5 (83–95%) was identified in the rSsa2p monomer peak either with or without nucleotides (data not shown). Thus, the monomeric form of Ssa2p is most favourable for Hst 5 binding, consistent with previous reports that hsc70 monomers favour protein or peptide substrate interactions (Gao *et al*., 1996). Because we were interested in total binding affinity under different conditions, polymer and monomer peaks were considered together to simplify calculation of dissociation constants (*K*_d_). The *K*_d_ for rSsa2p–BHst 5 complexes under each nucleotide condition were calculated using average values obtained from free Hst 5, co-eluted Hst 5 and Ssa2p peaks from at least three independent experiments (Table 1). Hst 5 was found to bind Ssa2p with *K*_d_ = 5.4 μM in the absence of added nucleotide, confirming that Ssa2p–Hst 5 complexes can be formed without nucleotides or cofactor proteins. However, addition of ATP or ADP reduced the *K*_d_ by about 10-fold, showing that ATP or ADP association with Ssa2p favours Hst 5 binding, but that Hst 5 does not compete for nucleotide binding sites in Ssa2p and further confirming that the binding site is outside the nucleotide binding groove. In addition, replacing ATP with non-hydrolysable ATPγS reduced the *K*_d_ by only threefold, also showing that ATP hydrolysis is not involved in formation of Ssa2-Hst 5 complexes. Thus, mutations within the Ssa2_334−338_ region (magenta) likely effect overall conformation of the ATPase domain resulting in alteration of Hst 5 binding to the Ssa2_128−135_ epitope.

**Table 1:** Nucleotides enhance binding of rSsa2p with BHst 5.

Nucleotide conditions	*K*_d_ (μM)
No nucleotide	5.4 ± 0.3
ATP (1 mM)	0.4 ± 0.1
ADP (0.9 mM) + ATP (0.1 mM) and P_i_ (1 mM)	0.6 ± 0.2
ATPγS (1 mM)	1.3 ± 0.4

rSsa2p (5 μM) was mixed with excess BHst 5 (100 μM) without or with 1 mM ATP-Mg^2+^, 0.9 mM ADP-Mg^2+^, 0.1 mM ATP-Mg^2+^ and 1 mM P_i_, or 1 mM ATPγS-Mg^2+^ in total volume of 250 μl in Buffer A to form complex. The mixture was loaded onto a FPLC Superose 12 10/300 column to separate rSsa2p–BHst 5 complexes from free BHst 5. The concentration of BHst 5 and rSsa2p was measured by densitometric scanning against quantification standards. Dissociation constants (*K*_d_) of rSsa2p–BHst 5 complexes under each nucleotide condition were calculated using the equation *K*_d_ = [BHst 5_f_][rSsa2p_f_]/[rSsa2p–BHst 5].

## Discussion

*Candida albicans* contains abundant Ssa1 and Ssa2 proteins in the unconventional location of the cell wall, as well as being present in the cytosolic compartment. The functional role of these proteins (as well as other Hsp70 proteins) in the cell envelope is not understood, although they may serve as recognition sites for antibodies and may retain chaperone functions with extracellular proteins (Botzler *et al*., 1998). Classical chaperone activity of Hsp70 family members involves co-chaperones including Hsp40, Hsp90, Hsp104; and nucleotide-dependent binding and release of client proteins from the peptide-binding domain. In contrast, the antifungal protein Hst 5 has direct interactions with Ssa2p that occur in the ATPase domain outside the conventional Ssa2 peptide-binding domain. Consistent with such ‘ectopic’ binding, nucleotides are not required for binding, suggesting that Ssa2p should be capable of binding proteins within the environment of the yeast cell envelope *in vivo* where there is limited availability of nucleotides. Furthermore, candidal cell wall Ssa proteins are capable of functioning as chaperone or facilitating proteins for Hst 5 entry into yeast cells without requiring co-chaperones, even though cell surface localization of Hsp90 and Hsp104 in *C. albicans* has been reported (Pitarch *et al*., 2002; Urban *et al*., 2003). Similar non-conventional binding associations have been reported for bacterial proline-rich cationic peptides that bind the lid domain of DnaK (Kragol *et al*., 2001) and bacterial ClpB (similar to Hsp104 in yeast) that binds substrates within its nucleotide-binding domain (Schlieker *et al*., 2004). In mammalian cells, Hsp70 can also bind short peptide sequences (8–26 amino acids in length) (Grossmann *et al*., 2004) indicating its versatility in binding with smaller substrates.

Hst 5–Ssa2p interactions are novel in that they do not require conventional binding sites, co-chaperones or nucleotides for function. However, the presence of nucleotides does enhance Hst 5 binding, suggesting that ATP binding to Ssa2p alters the secondary (or tertiary) conformation of the Hst 5 binding site in a way that improves Hst 5 interactions. Crystal structure data of Hsc70 (having 75% identity with candidal Ssa2p) under conditions of bound and unbound ATP/ADP (Jiang *et al*., 2005) provide insight into the conformational changes that occur upon nucleotide binding. Treatment of Hsc70 with ATP or ADP showed that the two lobes of ATPase domain rotate into a more closed conformation (7.4° rotation towards each other) in the presence of nucleotide (Jiang *et al*., 2005). If candidal Hsp70 follows this structural model, the Ssa2_128−135_ region should be more highly exposed upon nucleotide binding making the site more accessible for Hst 5 binding. Addition of nucleotides to Hst 5 prior to incubation with cells did not alter its activity (data not shown) suggesting that ATP itself does not bind to Hst 5 but instead affects subsequent interactions with Ssa2p. However non-hydrolysable nucleotide ATPγS did not substantially alter binding between Ssa2p and Hst 5 when compared with ADP or ATP (Table 1), suggests that Hst 5 binding is improved by conformational changes of Ssa2p related to nucleotide binding rather than nucleotide hydrolysis.

Many cationic peptides are salt sensitive, and Hst 5 is among them (Dong *et al*., 2003; Jang *et al*., 2008). Electrostatic mapping of full-length Ssa2p showed no specific clustering of negatively charged amino acid residues either in the whole protein or within identified Hst 5 binding sites (data not shown), indicating that Hst 5–Ssa2p interactions are not simply a result of electrostatic interactions between this cationic peptide and negatively charged areas on Ssa2p. Furthermore, buffer conditions that disrupt Hst 5 binding to intact cells (Dong *et al*., 2003) did not affect binding interactions between rSsa2p and Hst 5 (Fig. 2). Since Ssa2p is located within the cell envelope rather than at the cell surface, we conclude that salt-sensitive interaction between Hst 5 and intact cells likely involves initial binding at the cell surface and precedes Hst 5 binding with Ssa2p. Potential candidates for these salt-sensitive interactions with Hst 5 are negatively charged glucans or polysaccharide components on the outer cell wall surface.

Ssa proteins do not contain a canonical secretion signal, yet are abundantly present at the yeast cell surface. Alternative mechanisms by which proteins lacking N-terminal signal peptides are secreted could involve pathways outside the endoplasmic reticulum-Golgi secretion process such as adhesion to secretory vesicles, entry into endosomal subcompartments, passive transfer across the cell membrane or substrate-specific recognition by a transporter (Nombela *et al*., 2006). Interestingly, construction of one of our substitution mutants Ssa2_(334−338A3)_ resulted in complete expression of Ssa2 protein in cytoplasm; however, its secretion to the cell wall was nearly abolished. Inspection of this region showed two potential O-linked glycosylation sites (STRIP) that were removed with three introduced amino acid substitutions. These results suggest that post-translational glycosylation of Ssa2p residues 334–338 is required for cell wall secretion, and further imply that endoplasmic reticulum-Golgi pathway are involved in this secretion process. Investigation of levels of cell wall Ssa proteins in *C. albicans* secretion mutants may provide further information about this process. This mutant also provides strong evidence that cell wall, but not cytosolic, Ssa2p is involved in efficient uptake and killing by Hst 5.

Our experiments using *C. albicans* cells with selected mutations within Ssa2p revealed one likely contiguous binding site for Hst 5 mapped to amino acid residues 128–135. However, we were unable to rule out a second binding site identified from thermolysin digestion experiments in regions 330–341, since all mutations constructed in this region resulted in loss of Ssa2p expression and/or stability. Three-dimensional modelling showed that this site is also surface localized and adjacent to the 128–135 epitope. Thus, this site could be an independent binding epitope or a distal part of one single region. Although this region could be involved solely in structural conformation of the Ssa2 protein needed for Hst 5 binding, our digestion experiments indicate this region is directly associated with Hst 5 peptide.

Although Ssa2p clearly plays an important role in intracellular translocation of cationic peptides, our results point to a facilitator function rather than being an actual transporter/importer since (i) Ssa2p deletion mutants have reduced but measurable uptake of Hst 5, and (ii) the requirement of cellular energy for peptide import (Xu *et al*., 1999) suggests that the actual import mechanism involves active transport or energy-dependent processes such as endocytosis. One possibility is that Ssa2p serves as a chaperone to transfer cell wall-bound peptides to a membrane permease that transports cationic peptides. *S. cerevisiae* uses at least four plasma membrane permeases, DUR3 and SAM3 (Uemura *et al*., 2007), AGP2 (Aouida *et al*., 2005) and GAP1 (Uemura *et al*., 2005), that catalyse the uptake of extracellular polyamines in an energy-dependent manner. In this regard, yeast two-hybrid screening of the *S. cerevisiae* proteome for Hsp90 interactions uncovered strong interactions with a large group of membrane transporters including AGP2 (Millson *et al*., 2005), suggesting that these chaperones could function to pass substrates to transporter proteins. Candidal homologues of these permeases (*AGP2*, *GAP1*, *DUR3*) are potential candidates as intracellular transporters of Hst 5. Alternatively, *C. albicans* Ssa proteins have been identified in lipid rafts (Insenser *et al*., 2006), putting forth the possibility that Ssa2p and its client substrate are internalized by endocytosis. Thus, Hst 5 or other cationic peptides could be taken up by Ssap-mediated endocytosis then released into the cytosol by retrograde transport as has been described for yeast killer toxins in *S. cerevisiae* (Eisfeld *et al*., 2000; Breinig *et al*., 2006). Evidence for involvement of endocytotic processes has been reported for branched Hst 5 analogues as well as the finding that Hst 5 killing was inhibited by pre-treatment of cells with drugs blocking endocytosis (Zhu *et al*., 2006).

Our finding that Hst 5 binds to specific regions within candidal Ssa2p that are not homologous to human Hsp70 sites (5 out of 12 amino acids in the T1 region are different from human Hsc70 and Ssa2, alignment data not shown) identifies one reason why Hst 5 can kill *Candida* cells without being toxic to human tissues. This translocation mechanism is at least partially shared by other cationic peptides. For example, human β defensin 2 and 3 can kill candidal cells utilizing a similar uptake mechanism involving Ssa proteins (Vylkova *et al*., 2006). Further mapping of these binding interactions may be a basis for design of ‘smart’ peptides targeted to specific pathogenic fungal or bacterial cell wall proteins that allow rapid and selective eradication of these pathogens.

## Experimental procedures

### Peptides

Hst 5, inhibitor peptides (EEVD, EPSNDGPTVEEVD) within the C-terminus ‘anchor region’ and peptides Ssa2_127−157_, Ssa2_329−356_, Ssa2_68−83_ and Ssa2_245−257_ covering Hst 5 binding regions identified by peptide arrays for competition assays were synthesized by Genemed Synthesis. Hst 5 was biotinylated solely at its N-terminus. Biological activity of BHst 5 was verified by candidacidal assay as previously described (Edgerton *et al*., 1998).

### Plasmids

The DNA fragments encoding the *C. albicans* full-length Ssa2 protein (amino acid residues 1–645), the Ssa2p lacking the putative anchor domain (Ssa2_1−630_), the Ssa2p ATPase domain (Ssa2_1−385_) and the Ssa2p peptide-binding domain (Ssa2_386−645_) were produced by polymerase chain reaction and inserted into the BamHI and NotI cloning sites of the *S. cerevisiae* yeast expression vector pYES2/NT/C (Invitrogen) that contains polyhistidine and Xpress tags at the 5′ end and a galactose-inducible promoter.

### Production of recombinant proteins

Plasmids of Ssa2p and its truncated variants were transfected into competent INVSc-1 *S. cerevisiae* cells and expression was induced by galactose as previously described (Li *et al*., 2006). Cells were collected and re-suspended in ice-cold His-Binding Buffer (Zymo Research) supplemented with protease inhibitors then glass bead disrupted. Clarified cell lysates were collected following centrifugation at 13 000 *g* for 15 min. The clarified supernatant was loaded onto a His-Spin Protein Miniprep column (Zymo Research) and recombinant proteins were purified according to manufacturer's instructions. Purified proteins were pooled and dialysed at 4°C against Buffer A (20 mM HEPES, 3 mM KOH, 25 mM NaH_2_PO_4_, 25 mM Na_2_HPO_4_, 25 mM NaCl, pH 7.4) in D-Tube Dialyser Midi, MWCO 6–8 kDa (Novagen) for 3 h. Protein concentrations were tested on BCA Protein Assay Kit (Pierce) and 1 μg of each recombinant protein was loaded onto SDS-PAGE gel, transferred to PVDF membranes and Coomassie blue-stained to verify protein size and purity.

### Pull-down assays of truncated rSsa2p–BHst 5 complexes

BHst 5 (41 μg) was combined with purified rSsa2p, rSsa2_1−630_, rSsa2_1−385_ or rSsa2_386−645_ (10 μg) in binding Buffer B (0.1 M Phosphate, 0.15 M NaCl, pH 7.2), containing 6 mM Mg^2+^, and incubated for 2 h at 4°C to allow complex formation. For cross-linking experiments, 2.6 mM 3,3′-dithiobis-sulfosuccinimidylpropionate (DTSSP) (Pierce) was added to the incubation mixture to stabilize truncated rSsa2p–BHst 5 complexes. This cross-linker is a homobifunctional, thiol-cleavable, primary amine-reactive molecule with a long spacer arm (12 Å) designed for intermolecular cross-linking. Cross-linking stop solution (40 mM Tris, pH 7.5) was added to the mixture and incubated for 15 min. The reaction mixture was combined with immobilized Streptavidin-agarose beads (50 μl) (Pierce) for 1 h at 4°C, then washed and eluted as previously described (Li *et al*., 2006). Control reactions were performed with each truncated protein and Streptavidin-agarose beads alone in identical pull-down assay conditions. Captured proteins were subjected to 7.5% SDS-PAGE, and recombinant Ssa2 proteins were detected by Western blotting with Anti-Xpress-HRP monoclonal antibody (Invitrogen) and enhanced chemiluminescence (GH Healthcare) development. For competition experiments, C-terminal fragments (EEVD and EPSNDGPTVEEVD) were synthesized. A sixfold molar excess (4 mM) relative to full-length Ssa2p of each synthetic peptide was incubated with BHst 5 for 30 min at 4°C prior to addition of rSsa2p. Pull-down assays were then carried out using cross-linker as described above.

### Detection of rSsa2p–BHst 5 binding sites by limited proteolysis

Purified rSsa2p was extensively dialysed against Buffer A to remove any protease inhibitors remaining from purification. Optimal digestion conditions for rSsa2p were first established by assessment of the extent of rSsa2p digestion by titrating thermolysin concentration (0.2, 2, 5, 10 μg ml^−1^) over 0, 5, 30, 60 min incubation at 30°C. The rSsa2p (10 μg) was incubated with BHst 5 (20 μg) in the presence of ATP-Mg^2+^ (2 mM) for 30 min at room temperature with the addition of cross-linker (DTSSP). Stabilized rSsa2p–BHst 5 complexes were isolated by pull-down with Streptavidin-agarose beads. Beads were washed three times with Buffer B, then re-suspended in digestion buffer (50 mM Tris, pH 7.5, 150 mM KCl, 20 mM MgCl_2_) and subjected to thermolysin (5 μg ml^−1^) digestion at 30°C for 1 h with gentle shaking to ensure that beads remained suspended in digestion buffer. Digestion fragments not associated with the rSsa2p–BHst 5 complex binding site were removed by washing, and then the remaining bead-retained rSsa2p–BHst 5 complex was recovered. Pull-down fragments eluted from the beads were subjected to 15% SDS-PAGE and detected by silver staining (Sigma). Control thermolysin digestions of rSsa2p without addition of BHst 5 were loaded directly on the gel in parallel. The smallest major digestion fragments (≈ 8 kDa) released from Hst 5–Ssa2p complexes were selected for analysis by mass spectrometry analysis (Yale Cancer Center Mass Spectrometry Resource and W.M. Keck Foundation Biotechnology Resource Laboratory).

### Mapping of BHst 5-binding motifs on Ssa1/2 peptide arrays

Primary sequences of *C. albicans* Ssa1p and Ssa2p were obtained from the Universal Protein Resource (http://www.ebi.uniprot.org/) and peptide arrays of each Ssa1 and Ssa2 proteins were designed so that each peptide fragment consisted of sequential 13 amino acid fragments with an overlap of two amino acids between each peptide (*Supporting information*, Data S1). Overlapping peptides comprising the entire sequence of Ssa1p (656 amino acids) and Ssa2p (645 amino acids) were synthesized by JPT Peptide Technologies GmbH (Berlin, Germany) and printed on cellulose-β alanine membranes. The preparation of the membrane, as well as control experiments to detect false-positive binding of Streptavidin-HRP to the membrane, was performed following the manufacturer's protocol. Spot array cellulose-β alanine membranes were rinsed with methanol for 5 min, followed by three washes with 20 ml of TBS (50 mM Tris-HCl, 137 mM NaCl, 2.7 mM KCl, pH 8.0) for 10 min. The membrane was then blocked with Starting Block (Pierce) blocking buffer (an albumin and endogenous biotin-free buffer compatible with a Streptavidin system) for 2 h at room temperature. BHst 5 (2 μM) was added to the blocking buffer and incubated with the membrane for 3 h at room temperature. After extensive washing with TBS buffer to remove unbound BHst 5, the membrane was incubated with Streptavidin-HRP (1:20 000) for 1 h at room temperature. After washing six times with TBS buffer, membranes were developed with SuperSignal West Pico Chemiluminescent Substrate (150 μl cm^−2^) (Pierce) for 5 min and scanned using a Fuji LAS-1000 Plus Chemiluminescence Imager, and signals were quantified using ImageJ software (NIH). Repeatability of binding signals was confirmed by reprobing each membrane following regeneration with regeneration buffer (62.5 mM Tris-HCl, 2% SDS, 0.7% 2-mercaptoethanol, pH 6.7) for 30 min at 50°C. The strongest spot signals from each array were aligned with primary sequences of Ssa1 and Ssa2 proteins.

### Site-directed mutagenesis

Since the immediate upstream 1736 bp of the *SSA2* coding sequence lacks a stress response element, we could not express Ssa2 protein using this region as a promoter in RP10 locus of *ssa2*Δ strain (Li *et al*., 2006). Therefore, the *SSA1* promoter region (965 bp immediate upstream of *SSA1* coding sequence) was cloned into PstI*/*SalI digestion sites of pDBU3R (Li *et al*., 2006) to drive *SSA2* gene expression. The *SSA2* coding sequences (1938 bp) and immediate downstream untranslated region (3′ UTR) 582 bp were cloned to the SalI*/*BamHI sites of the same vector (primers were listed in Table 2). This plasmid was used as a template for site-directed mutagenesis of Hst 5 binding areas identified through *in vitro* assays. Alternative amino acids (3–4) from the putative binding sites were replaced with Ala using PAGE-purified mutagenesis primers (Table 2). For quick selection of the correctly mutated plasmids, specific restriction enzyme cutting sites were considered during the design of the mutagenesis primers (Table 2). Mutagenesis PCR reactions were carried out using the Quick Change II XL Site-Directed Mutagenesis Kit (Stratagene) following the manufacturer's guidelines. Final plasmid constructs were sequenced to confirm mutations. Sequence analysis of the *C. albicans SSA2* coding region showed no CTG codons that could be miscoded in a *S. cerevisiae* recombinant protein expression system.

**Table 2:** Site-directed mutagenesis primers.

Primers	Sequences (5′ to 3′)	Digestion site
*SSA1* promoter-f	aag-CTGCAG-agtattgtagaataaattattcagc	PstI (CTGCAG)
*SSA1* promoter-r	aag-GTCGAC-aatttaattttttgtttaattgtgtttt	SalI (GTCGAC)
*SSA2* CDS + 582 bp UTR-f	aag-GTCGAC-atgtctaaagctgttggtattga	SalI (GTCGAC)
SSA2 CDS + 582 bp UTR-r	aag-GGATCC-atccaatcataatccagtaaattg	BamHI (GGATCC)
Ssa2_128−132A3_	gatcttgggtaaaatgaaggaa**g**ccg*CT****G****CAG*gt**gc**cttgggtaccac	PstI (CTGCAG)*
Ssa2_131−135A3_	aaatgaaggaaaccgctg*AAG****C****TT*tc**gc**gggt**g**ccactgttaaagatgctgttgtc	HindIII (AAGCTT)*
Ssa2_150−156A4_	actgtcccagcttacttcaatg**c**ttcc**gc**aaga**g*C****AGCT****G***ccaaagatgctggtaccattgc	PvuII (CAGCTG)*
Ssa2_334−338A3_	caaatctaaagttgatgaaattg***C****CTTGG***c**tggtg**c**ttctaccagaattccaaaggttc	StyI (CCTTGG)*

Sequences of sense mutagenesis primers for each construct are shown. Each PstI digestion site (CTGCAG), SalI digestion site (GTCGAC) and BamHI digestion site (GGATCC) in the template primers are capitalized. All mutated nucleotides are indicated by bold underline. New digestion sites (*) generated by mutation and used for rapid colony selection are indicated by capitals and italics. UTR, 3′ untranslated regions.

### Mutant strain construction and mutant protein expression

All plasmids were linearized with NocI enzyme and transformed to the *ssa2*Δ strain (Li *et al*., 2006). Correct integration of each plasmid to the RP10 locus was verified by PCR. CAF4-2 (wt), *ssa2*Δ, *ssa2*Δ*/SSA2*, and additional *SSA2* mutant strains (Ssa2_(128−132A3)_, Ssa2_(131−135A3)_, Ssa2_(150−156A4)_, Ssa2_(334−338A3)_) were grown in (yeast extract/peptone/dextrose) YPD medium with uridine (50 μg ml^−1^) at 30°C overnight, and collected at OD_600_ = 1.8. To detect protein expression levels, cells were washed twice with 10 mM sodium phosphate buffer (NaPB), and cell wall or cytosolic fractions were collected as described (Li *et al*., 2006). Total proteins (25 μg) from each sample were subjected to 7.5% SDS-PAGE, immunoblotted using Hsp70/Hsc70 monoclonal antibody (SPA-822, StressGen Biotechnologies), and detected by ECL (Amersham Pharmacia Biotech).

### Candidacidal assays of Hst 5

Susceptibilities of *Candida* wt and *SSA2* mutant strains to Hst 5 were examined by microdilution assays (Dong *et al*., 2003) with the following modifications. Strains were grown overnight in YPD medium, washed twice with 10 mM NaPB and re-suspended to 10^6^ cells ml^−1^. Cells were then incubated with 15 μM Hst 5 for 1 h at 30°C, with gentle rotation (250 r.p.m.). Cell suspensions were diluted and aliquots of 500 cells were spread onto YPD agar plates and incubated for 48 h at room temperature and the percentage cell death compared with cells treated with buffer only was calculated. For each mutant strain, three independent transformant colonies were selected for testing and each assay was repeated at least three times.

### Hst 5 translocation assays

Intracellular translocation of BHst 5 in *Candida* wt and *SSA2* mutant strains was tested as we have previously described (Jang *et al*., 2008). Briefly, early log-phase cells (1 × 10^8^) were washed twice with 10 mM NaPB, suspended in 1 ml of NaPB, and BHst 5 was added to a final concentration of 15 μM and incubated at room temperature for 30 min. Translocation happens very rapidly following only 5 min incubation with Hst 5; while the majority of Hst 5 is translocated intracellularly after 30 min. Following incubation, excess extracellular Hst 5 was removed by washing, and cells were collected at 3 300 *g* at 4°C. Cytosolic Hst 5 was collected together with cytosol proteins after disruption with glass beads using a FASTPREP®-24 (MP) and centrifugation at 15 700 *g* for 10 min. Protein concentration for each preparation was measured by BCA assay (Pierce) and cytosolic proteins (10 μg) were separated using 16% Tricine SDS-PAGE, immunoblotted with Streptavidin conjugated with horseradish peroxidase (Pierce), and developed by ECL. Intracellularly translocated Hst 5 was quantified using Quantity One software (Bio-Rad). The reported values are means ± standard errors of triplicate independent assays for each cell type.

### Construction of predicted secondary and tertiary structures of Ssa2p

Ssa1 and Ssa2 proteins sequence alignments were performed using clustal x (1.83) multiple sequence alignment software (http://bips.u-strasbg.fr/en/Documentation/ClustalX/). Ssa2p–Hst 5 interaction epitopes identified from peptide arrays (blue) and limited digestion (green) were mapped onto aligned sequences of the Ssa2p ATPase domain, and overlapping areas detected using both methods were indicated with red boxes. The secondary structure of Ssa2p was predicted from the protein homology analogy recognition engine (http://www.sbg.bio.ic.ac.uk/~phyre/). Tertiary structure of full-length *C. albicans* Ssa2p was predicated from ROBETTA Full-chain Protein Structure Prediction Server (http://robetta.bakerlab.org/). Epitopes that had strong effects on Hst 5 function as assessed by mutational analyses (Ssa2_128−135_) were labelled with green, while the area representing a possible functional site (Ssa2_334−338_) was labelled with magenta using the PyMOL Molecular Graphics System 2002 (http://www.pymol.org) on ribbon and surface models of Ssa2p structures.

### FPLC gel filtration determination of dissociation constants

Superose 12 10/300 GL AKTA (GE Healthcare) FPLC was chosen for separation of rSsa2p (78 kDa) from BHst 5 (3 kDa). Purified rSsa2p (5 μM) was mixed with excess BHst 5 (100 μM) with or without different nucleotides. Nucleotides added were one of the following: 1 mM ATP-Mg^2+^ 1 mM ATPγS-Mg^2+^ or 0.9 mM ADP-Mg^2+^, 0.1 mM ATP-Mg^2+^ and 1 mM P_i_ (Greene *et al*., 1995). The total volume was adjusted to 250 μl with the same dialysis buffer used for purification of rSsa2p (Buffer A), and the protein mixture was incubated for 30 min on ice before loading onto Superose 12 10/300 GL FPLC column equilibrated in Buffer A or Buffer A containing nucleotides at a flow rate of 0.25 ml min^−1^. Fractions (0.25 ml) were collected, 25 μl of each fraction around the peak area was loaded on 7.5% SDS-PAGE gel and silver-stained to locate rSsa2p-positive fractions (Fig. 9, left). Pure rSsa2p and BHst 5 were loaded in gradient onto the Minifold II Slot Blot System (Whatman) to obtain a linear standard range (Fig. 9, right, lane 1 and 2). Fractions containing rSsa2p was pooled, and a concentration range of pool A and pool B were loaded together with each standard and detected with anti-Xpress or Streptavidin-HRP antibodies for rSsa2p or BHst 5 respectively. Only proteins falling within the linear range were used for quantification by densitometric scanning (Bio-Rad) against the quantification standards. Dissociation constants (*K*_d_) of rSsa2p–BHst 5 complexes under each nucleotide condition were calculated using the equation *K*_d_ = [BHst 5_f_][rSsa2p_f_]/[rSsa2p–BHst 5] (Kim and McHenry, 1996) to compare the effects of each nucleotide on binding affinities.
