# Supplementary material for: Uptake of the antifungal cationic peptide Histatin 5 by Candida albicans Ssa2p requires binding to non-conventional sites within the ATPase domain
Source: Mol Microbiol. 2008 Oct 20;70(5):1246–60. doi: 10.1111/j.1365-2958.2008.06480.x (PMC2643122; doi:10.1111/j.1365-2958.2008.06480.x)
Supplement: Supplementary file 1 [file mmi0070-1246-SD1.pdf]

Data S1

N-terminus: acetyl

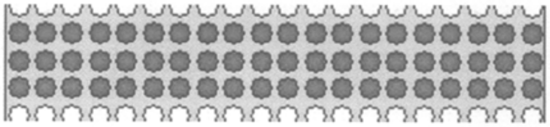

Ssa1 array

1	SKAVGIDLGTTYS	16	VLRIINEPTAAAI	31	EIVLVGGSTRIPK	46	TQKITITNDKGRL
2	YSSVAHFANDRVE	17	AIAYGLDKKGSRG	32	PKIQKLVSDFFNG	47	RLSKEEIDKMVSE
3	VEIIANDQGNRTT	18	RGEHNVLIFDLGG	33	NGKELNKSINPDE	48	SEAEKFKEEDEKE
4	TTPSFVAFTDTER	19	GGGTFDVSLLAID	34	DEAVAYGAAVQAA	49	KEAARVQAKNQLE
5	ERLIGDAAKNQAA	20	IDEGIFEVKATAG	35	AAILTGDTSSKTQ	50	LESYAYSLKNTIN
6	AAMNPANTVFDAK	21	AGDTHLGGEDFDN	36	TQDILLLDVAPLS	51	INDGEMKDKIGAD
7	AKRLIGRKFDDPE	22	DNRLVNFFIQEFK	37	LSLGIETAGGIMT	52	ADDKEKLTKAIDE
8	PEVINDAKHFPFK	23	FKRKNKKDISTNQ	38	MTKLIPRNSTIPT	53	DETISWLDASQAA
9	FKVIDKAGKPVIQ	24	NQRALRRLRTASE	39	PTKKSETFSTYAD	54	AASTEEYEDKRKE
10	IQVEYKGETKTFS	25	SERAKRTLSSSAQ	40	ADNQPGVLIQVFE	55	KELESVANPIISG
11	FSPEEISSMVLTK	26	AQTSIEIDSLYEG	41	FEGERAKTKDNNL	56	SGAYGAAGGAPGG
12	TKMKEIAEGYLGS	27	EGIDFYTSITRAR	42	NLLGKFELSGIPP	57	GGAGGFPGAGGFP
13	GSTVKDAVVTVPA	28	ARFEELSADLFRS	43	PPAPRGVPQIEVT	58	FPGGAPGAGGPGG
14	PAYFNDSQRQATK	29	RSTLDPVGKVLAD	44	VTFDIDANGILNV	59	GGATGGESSGPTV
15	TKDAGTIAGLNVL	30	ADAKIDKSQVEEI	45	NVSALEKGTGKTQ	60	GGESSGPTVEEVD

N-terminus: acetyl

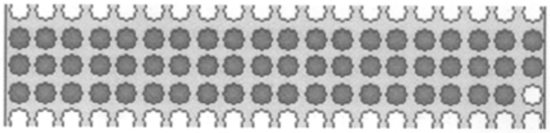

Ssa2 array

1	SKAVGIDLGTTYS	16	VMRIINEPTAAAI	31	IVLVGGSTRIPKV	46	QKITITNDKGRLS
2	YSSVAHFANDRVE	17	AIAYGLDKKSEAE	32	KVQKLVSDYFNGK	47	LSKEEIEKMVSEA
3	VEIIANDQGNRTT	18	AEKNVLIFDLGGG	33	GKEPNRSINPDEA	48	EAEKFKEEDEKEA
4	TTPSFVAFTDTER	19	GGTFDVSLLSIED	34	EAVAYGAAVQAAI	49	EASRVQAKNQLES
5	ERLIGDAAKNQAA	20	EDGIFEVKATAGD	35	AILSGDTSSKTQD	50	ESYAYSLKNTLGE
6	AAMNPANTVFDAK	21	GDTHLGGEDFDNR	36	QDLLLLDVAPLSL	51	GEEQFKSKLDASE
7	AKRLIGRKFDDHE	22	NRLVNFFIQEFKR	37	SLGIETAGGIMTK	52	SEIEEVTKAADET
8	HEVQGDIKHFPFK	23	KRKNKKDISTNQR	38	TKLIPRNSTIPTK	53	ETIAWLDSNQTAT
9	FKVVDKASKPMIQ	24	QRALRRLRTASER	39	TKKSETFSTYADN	54	ATQEEFADQQKEL
10	IQVEYKGETKTFS	25	ERAKRTLSSSAQT	40	DNQPGVLIQVFEG	55	ELESKANPIMTKA
11	FSPEEISSMILGK	26	QTSIEIDSLYEGI	41	EGERAQTKDNNLL	56	KAYQAGATPSGAA
12	GKMKETAEGFLGT	27	GIDFYTSITRARF	42	LLGKFELSGIPPA	57	AAGAAPGGFPGGA
13	GTTVKDAVVTVPA	28	RFEELSADLFRST	43	PAPRGVPQIEVTF	58	GAAPEPSNDGPTV
14	PAYFNDSQRQATK	29	STLEPVDKVLSDA	44	TFDIDANGILNVS	59	EPSNDGPTVEEVD
15	TKDAGTIAGLNVM	30	DAKIDKSKVDEIV	45	VSALEKGTGKTQK		
